# Melanonychia

**Published:** 2015-08-20

**Authors:** Michael J. Metzner, Alicia R. Billington, Wyatt G. Payne

**Affiliations:** Plastic Surgery Section, Bay Pines VA Healthcare System, Bay Pines, Fla; and Division of Plastic Surgery, University of South Florida College of Medicine, Tampa

**Keywords:** melanonychia, subungual melanoma, nail, hyperpigmentation, melanocyte

## DESCRIPTION

A 60-year-old white woman presented with a 1-year history of melanonychia of the left small fingernail, which she hid with nail polish. There were multiple hyperpigmented bands longitudinally oriented. Of note, the patient had a history of skin cancer and a family history of melanoma.

## QUESTIONS

1. **What is the epidemiology of melanonychia?**
2. **What is the pathophysiology of melanonychia?**
3. **What evaluation options are available for the diagnosis of melanonychia?**
4. **What are the treatment options for melanonychia?**

## DISCUSSION

**Figure 1:**
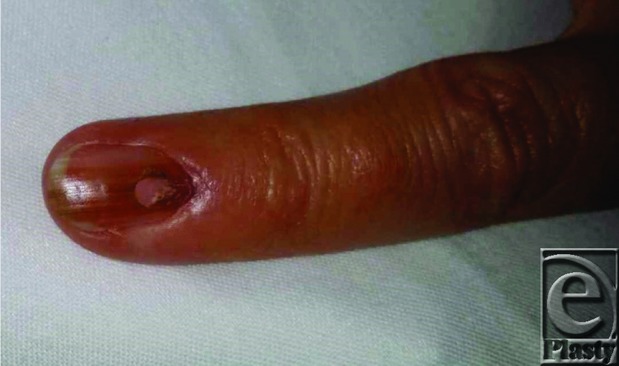
Melanonychia of the small finger of the left hand with biopsy site from trephination. Biopsy was inconclusive, and more invasive biopsy was warranted.

Melanonychia is characterized as a brown or black hyperpigmentation of the nail. The band usually presents lengthwise along the nail unit and is also known as longitudinal melanonychia (LM) or melanonychia striata (Fig 1). It is important that clinicians understand the epidemiology, pathophysiology, assessment, and treatment options for melanonychia, as the causes range from the more common benign causes to the less common aggressive subungual melanoma. Melanonychia is more frequent in darker pigmented and older individuals. Longitudinal melanonychia is found in 77% of the black population by 20 years of age and almost 100% of individuals older than 50 years.^1^ In a study of Chinese patients, melanonychia affected males and females equally, with the morbidity and mortality dependent on the underlying cause of the condition.^2^

There are many causes of melanonychia, and the presence of LM can indicate both local and systemic diseases. The major pathophysiological pathways include melanocytic activation, hyperplasia, or the invasion of the nail matrix by a melanin-producing pathogen. Melanocytic activation occurs when melanocytes found within the nail matrix are stimulated to produce pigment, or melanin, without an increase in the total number of cells.^3^ Since melanocytes are mostly dormant in the proximal nail matrix, most melanonychia usually originates from active melanocytes in the distal nail matrix.^4^ Melanocytes can be activated through various physiological, local, regional, dermatological, iatrogenic, and syndromic processes. Melanocytic hyperplasia occurs with an increase in the number of melanocytes, thus leading to more melanin production.^3^ The increase in number of melanocytes can be indicative of a nevus or melanoma. Unfortunately, because of the common misdiagnosis of nail apparatus melanoma, there is an average delay of diagnosis by 2 years, leading to a poor prognosis with reported 5-year and 10-year survival rates of only 30% and 13%, respectively.^5^

**Figure 2:**
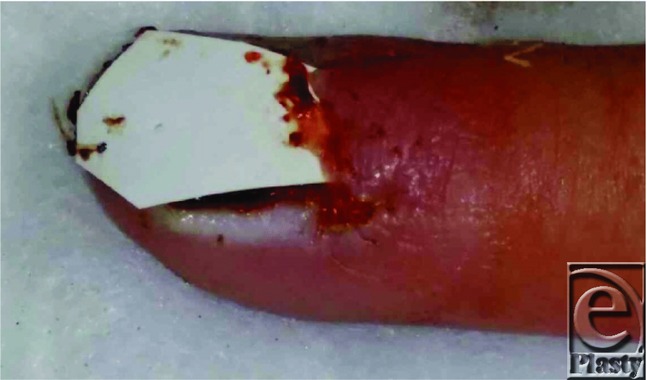
Complete nail plate removal for biopsy with insertion of sterile silicone sheeting in nail fold to prevent synechiae.

The proper evaluation and testing of melanonychia are important to quickly rule out dangerous pathologies. Clinical guidelines to assess LM were created following the ABCDEF pneumonic. A stands for age and race, B stands for brown or black band or breadth in the nail bed, C stands for change in band morphology, D stands for digit(s) involved, E stands for extension (Hutchinson's sign), and F stands for family history of melanoma.^6^ Hutchinson's sign is pigmentation extending over the proximal or lateral nail and is an important, although not absolute, sign of subungual melanoma. Biopsy and nail-clipping culture should be performed to further evaluate the underlying cause of melanonychia. The biopsy site should be chosen on the basis of the origin of the pigmentation, with the majority of LM cases originating distally. Depending on the width and location of hyperpigmentation, multiple techniques including punch, matrix shave biopsy, and lateral longitudinal excision of the nail matrix are treatment options.^7^ Figure 2 shows removal of the nail to allow for diagnosis following inconclusive punch biopsy.

Treatment of melanonychia depends on the underlying pathology. Treating the underlying systemic disease, removing an offending drug, or using antibiotic or antifungal treatments may cause the hyperpigmentation to regress. In the majority of cases, benign causes do not necessitate treatment. In the cases of subungual melanoma (depending on the thickness and invasive characteristics of the malignancy found after biopsy), wide local excision, amputation, and sentinel lymph node mapping/biopsy may be indicated.^8^

Although the majority of melanonychia cases have benign causes, it is crucial for clinicians to quickly identify the underlying cause to rule out malignancy. In the case of our patient, a nail bed biopsy and fingernail specimen showed fibrovascular tissue with chronic inflammation and scattered foreign body giant cells. A Grocott's methenamine silver stain showed scattered fungal elements consistent with an onychomycosis. The patient was treated with antifungals and the melanonychia resolved.

## Figures and Tables

**Figure 3:**
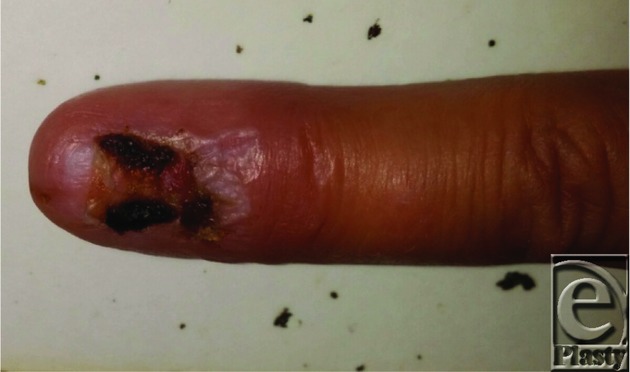
Postoperative week 2.

**Figure 4:**
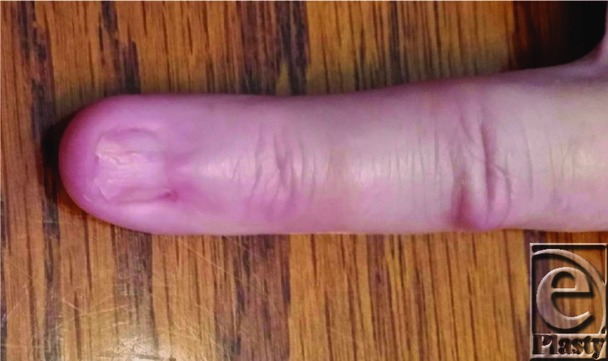
Postoperative month 6
